# Genetic variants differentially associated with rheumatoid arthritis and systemic lupus erythematosus reveal the disease-specific biology

**DOI:** 10.1038/s41598-019-39132-2

**Published:** 2019-02-25

**Authors:** Jiwoo Lim, Kwangwoo Kim

**Affiliations:** Department of Biology, Kyung Hee University, Seoul, Republic of Korea

## Abstract

Two rheumatic autoimmune diseases, rheumatoid arthritis (RA) and systemic lupus erythematosus (SLE), have distinct clinical features despite their genetic similarities. We hypothesized that disease-specific variants exclusively associated with only one disease could contribute to disease-specific phenotypes. We calculated the strength of disease specificity for each variant in each disease against the other disease using summary association statistics reported in the largest genome-wide association studies of RA and SLE. Most of highly disease-specific associations were explained by non-coding variants that were significantly enriched within regulatory regions (enhancers or H3K4me3 histone modification marks) in specific cell or organ types. (e.g., In RA, regulatory T primary cells, CD4+ memory T primary cells, thymus and lung; In SLE, CD19+ B primary cells, mobilized CD34+ primary cells, regulatory T primary cells and monocytes). Consistently, genes in the disease-specific loci were significantly involved in T cell- and B cell-related gene sets in RA and SLE. In summary, this study identified disease-specific variants between RA and SLE, and provided statistical evidence for disease-specific cell types, organ and gene sets that may drive the disease-specific phenotypes.

## Introduction

Rheumatoid arthritis (RA) and systemic lupus erythematosus (SLE) are well-characterized rheumatic autoimmune diseases with >60% heritability^1,2^. Genome-wide association studies (GWAS) have uncovered the highly polygenic etiology of RA and SLE, bringing the known disease-associated loci to about 100 in each disease^3–7^. The majority of the identified genetic associations are explained by common-frequency variants with modest effect sizes.

A dozen same disease alleles have been detected at the genome-wide significance level in both RA and SLE^3,4^. For examples, some variants in immune-related genes such as *HLA-DRB1*, *PTPN22*, *STAT4*, and *TNFAIP3* were reported to contribute to risk of RA and SLE^8–11^. These pleiotropic variants associated with both RA and SLE strongly suggest that the pathogenesis leading to immune dysfunction is partially shared between these two autoantibody-producing diseases. Evidence of pleiotropic variants in similar diseases motivated cross-disease meta-analyses^12,13^ and phenome-wide association studies to identify pleiotropic variants that explain common pathogenesis in different diseases.

Although RA and SLE share some genetic etiologies, they have highly distinct clinical features in terms of primarily inflamed sites, disease prognosis, and autoantigens. Thus, it is tempting to hypothesize that disease-specific variants exclusively associated with only one disease drive disease-specific features of the disease.

Here, we comprehensively investigated the dissimilarity in disease-variant associations between RA and SLE at the genome-wide level, and identified highly disease-specific variants that map to disease-specific cell types and pathways.

## Methods

### GWAS summary association statistic data

The largest-ever European GWAS summary association statistic data for RA and SLE were obtained from Okada *et al*.^3^ and Bentham *et al*.^4^, respectively. Both datasets were generated after whole-genome imputation using the reference panel from the 1000 Genomes Project data^14^ followed by disease association tests using multivariable logistic regression adjusting for population stratifications. The summary statistics for disease association include odds ratios of effect alleles, their standard errors (or 95% confidence intervals), effect alleles, non-effect alleles, association *P* values, and imputation quality in autosomal single nucleotide polymorphisms (SNPs). All subsequent analyses were performed for the SNPs with minor allele frequency (MAF) > 0.5% and imputed score >0.5 in RA and/or SLE datasets.

### Meta-analysis

A cross-disease meta-analysis was performed using GWAMA software^15^, which calculate the inverse of variances to weight effect sizes, based on the fixed-effects model. The heterogeneity of association effect estimates between RA and SLE was assessed using Cochran’s Q test.

### Estimating the strength of disease specificity

We created a statistic (*S*) that indicates the strength of disease-specific association of a variant in a disease using the following equation:

$$Strength\,of\,disease\,specificity\,({S}_{ij})=|\frac{{\beta }_{ij}}{s{e}_{ij}}|\times {Z}_{i},$$

where *S*_*ij*_ is the disease specificity of SNP *i* in disease *j*; *β*_*ij*_ is the effect size of the effect allele in the SNP *i* in disease *j*; *se*_*ij*_ is the standard error of *β*_*ij*_; and *Z*_*i*_ is the *z*-score transformed from *P* values in Cochran’s Q test which indicates the effect-size heterogeneity of SNP *i* between RA and SLE. The *S*_*ij*_ statistic is calculated by multiplying the absolute value of a *t*-statistic (equal to *β*_*ij*_ divided by *se*_*ij*_) by *Z*_*i*_. Thus, the *S* statistic is synergistically increased only when the association of a tested SNP is strong in a disease and the effect estimates between two diseases are highly heterogeneous. The near-zero value of the *t*-statistic and/or *Z*-score results in weak or mild *S* estimates. Each variant generates two *S* statistics (each for each disease); we compared the two *S* statistics from each variant and used the larger *S* value for the subsequent analyses. Then, all variants were ranked by the strength of disease specificity for each disease. Finally, the top 1% of SNPs in each disease were extracted to use in all subsequent enrichment analyses for each disease.

### Enrichment analysis using H3K4me3 histone modification marks

An enrichment analysis was performed using the Epi-GWAS software^14,16^ to determine whether disease-specific variants significantly overlap with histone posttranslational modification marks in specific cell types. Among the various types of histone marks, H3K4me3 is well-known as the most cell-type specific histone modification mark^16^. Briefly, the enrichment score was calculated based on the positions of query and proxy SNPs (*r*^2^ > 0.8) within H3K4me3 peaks for each cell type (obtained from chromatin immunoprecipitation sequencing in the Roadmap Epigenomics Project)^17,18^, taking into account the height and position of peak summits. Then, a permutation test was performed to identify the cell types that exhibited significantly high overlap between the H3K4me3 marks and disease-specific variants.

### Enrichment analysis using enhancers

An enrichment analysis was also performed using enhancer annotation data for 71 cell types and 41 organ types obtained from the FANTOM5 consortium database^19,20^. An enhancer-by-cell type matrix or enhancer-by-organ matrix filled with 0 (=no enhancer) or 1 (=enhancer) was constructed, and the matrix values were randomly shuffled by cell or organ type 10,000 times, preserving the total number of the enhancers in each cell type or organ. The expected distribution of the numbers of enhancers and disease-specific SNPs in each cell or organ type was obtained by counting the shuffled enhancers in which the query and proxy (r^2^ > 0.8) SNPs were located. Thus, this procedure did not disrupt the important property such as the total number of enhancers in each cell type, the number of appearances of each enhancer across different cell types, and the linkage disequilibrium among the query SNPs. The significance value of overlaps in each of cell or organ types was calculated by determining the percentile rank of the actual number of the overlap between enhancers and disease-specific SNPs in the tested cell or organ types among the numbers of the overlaps observed from the 10,000 shuffled matrices.

### Gene set enrichment analysis

A gene set enrichment analysis was conducted using DEPICT software^14,21^ to identify biological pathways implicated by genes in disease-specific loci that were exclusively associated with only one disease. Briefly, the top 1% of disease-specific SNPs (pruned by r^2^ < 0.2 at *P* in a cross-disease meta-analysis <0.05) and their percentile ranking in each disease were used as query variants and *P* values, respectively. The DEPICT software uses the customized gene sets that were previously generated from existing gene set databases^22–26^ and the co-expression data from human microarrays^27^. Genes that were mapped with neighboring disease-specific SNPs in each disease were tested for enrichments in the customized biological pathways based on a false-discovery rate (FDR) threshold of 20%.

## Results

### Numerous disease-risk variants shared between RA and SLE

To explore the disease-specific associations between RA and SLE, we obtained the association summary statistics from the largest-ever GWAS in European populations. The study populations consisted of 43,923 controls and 14,361 cases in the RA GWAS, and 6,959 controls and 4,036 cases in the SLE GWAS. A total of 8,031,027 autosomal SNPs with MAF ≥ 0.5% and imputation quality score ≥0.5 in both RA and SLE datasets were analyzed in this study.

First, before focusing on association differences in RA and SLE, we evaluated a degree of risk-allele sharing between two diseases. To avoid inflated estimates of the association similarity or dissimilarity in two diseases due to multiple SNPs in linkage disequilibrium (LD), we extracted 369,955 SNPs that were not correlated with each other (r^2^ < 0.2) within a 2-Mb flanking window based on the genotypes in the European populations of the 1000 Genomes Projects. The SNP-disease association *P* values displayed a significantly positive correlation between RA and SLE in the logarithm scale with a plus or minus sign representing the positive or negative effect of a minor allele, respectively (*P* < 2.2 × 10^−16^; Supplementary Fig. S1). These results suggest that both diseases share a number of disease-risk variants.

**Figure 1:**
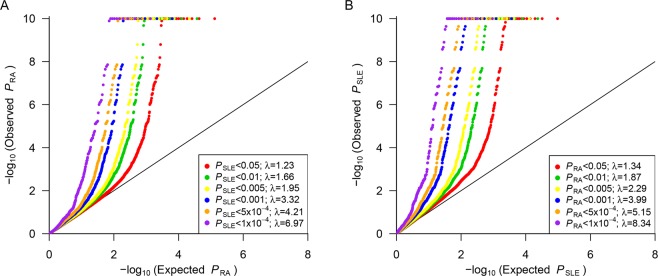
(**A**) Quantile-quantile plot showing inflated RA associations of SLE-associated SNPs with *P*_SLE_ < 0.05, 0.01, 0.005, 0.001, 5 × 10^−4^ or 1 × 10^−4^. (**B**) Quantile-quantile plot showing inflated SLE associations of RA-associated SNPs with *P*_RA_ < 0.05, 0.01, 0.005, 0.001, 5 × 10^−4^ or 1 × 10^−4^. The diagonal red line represents perfect concordance of observed and expected –log_10_*P* values.

We identified highly inflated associations of SNPs for one disease (either RA or SLE) that were associated with the other disease (either SLE or RA) at various significance threshold levels, as shown in conditional quantile-quantile plots^28^ in Fig. 1. For example, variants with an RA-association *P* value < 0.05 showed inflated SLE associations compared to those expected based on randomly selected SNPs (inflation factor λ = 1.34). When the same analysis was performed using variants with more significant *P* values in RA (e.g., *P* < 10^−4^), we found more inflated associations with SLE (λ = 8.34). Similarly, SLE variants passing various association *P *value thresholds were extracted and evaluated for their associations with RA. The results clearly indicated that the association with RA became highly inflated as the *P* value thresholds for SLE associations declined (Fig. 1). In addition, a pleiotropy analysis for all pruned SNPs with *r*^2^ < 0.2 in RA and SLE using GPA^29,30^ consistently supported a high degree of disease-allele sharing (*P* < 1.0 × 10^−100^).

We identified 14 non-HLA loci surpassing the genome-wide significance level in a cross-disease meta-analysis (2.43 × 10^−12^ ≤ *P*_meta_ ≤ 4.48 × 10^−8^) but not in a single disease association analysis (Supplementary Table S1). Of them, 13 regional associations were validated in additional samples in the same studies^3,4^ or recent independent studies^5,6,31,32^, which also indicates the RA and SLE have highly similar genetic architectures. (One locus with SNP rs8045689 that have not yet been significantly associated with RA or SLE could be a good candidate of a pleiotropic disease-associated variant).

### Identification of disease-specific loci

We extracted 64,845 SNPs from both the RA and SLE datasets that were not correlated with each other (*r*^2^ < 0.2). We calculated the strength of disease specificity (*S*) for each SNP using the disease-specific effect estimate, its standard error, and heterogeneity of effect estimates between two diseases (details in METHODS). Highly RA- and SLE-specific SNPs are listed in Supplementary Tables S2 and S3, respectively. For example, the SNP rs34185821 is located 80 kb from *RBPJ* and displayed one of the highest scores of disease specificity in RA (*P*_RA_ = 2.20 × 10^−16^ and OR_RA_ = 0.87 in RA; *P*_SLE_ = 0.419 and OR_SLE_ = 1.02 in SLE), whereas the intergenic SNP rs13019891 was highly SLE-specific (*P*_RA_ = 0.90 and OR_RA_ = 1.00 in RA; *P*_SLE_ = 2.26 × 10^−36^ and OR_SLE_ = 1.86 in SLE).

We selected the top 1% of the most disease-specific non-major histocompatibility complex (non-MHC) SNPs in RA and SLE, and then retrieved their proximal SNPs that were correlated with the disease-specific SNPs (*r*^2^ > 0.8 in European populations in the 1000 Genomes Project). Finally, a total of 4,913 RA-specific SNPs and 8,223 SLE-specific SNPs were used for all subsequent analyses. Most of these variants were found in non-coding regions, and 3.5% of loci (23 loci out of 648) had at least one leading or proxy variant that altered amino acid residues. Therefore, we postulated that the disease-specific SNPs drive the disease-specific phenotypes primarily by regulating gene expression rather than regulating protein activity.

### Specific cell or organ types implicated by disease-specific variants

As the majority of the disease-specific loci were explained by non-coding variants, we hypothesized that disease-specific allele affected the regulatory elements (e.g. histone marks and enhancers), which are highly cell type-specific. Under this assumption, we aimed to identify disease-relevant cell or organ types whose regulatory regions should be enriched with the disease-specific variants. We annotated each disease-specific variant and its proxy variants (*r*^2^ > 0.8) for the following potential regulatory elements: (1) the most cell type-specific histone modification marks (H3K4me3) in 34 cell types from the NIH Roadmap epigenomics consortium^17,18^ and (2) enhancer regions in 71 cell types and 41 organ types from the FANTOM5 consortium^19,20^.

**Figure 2:**
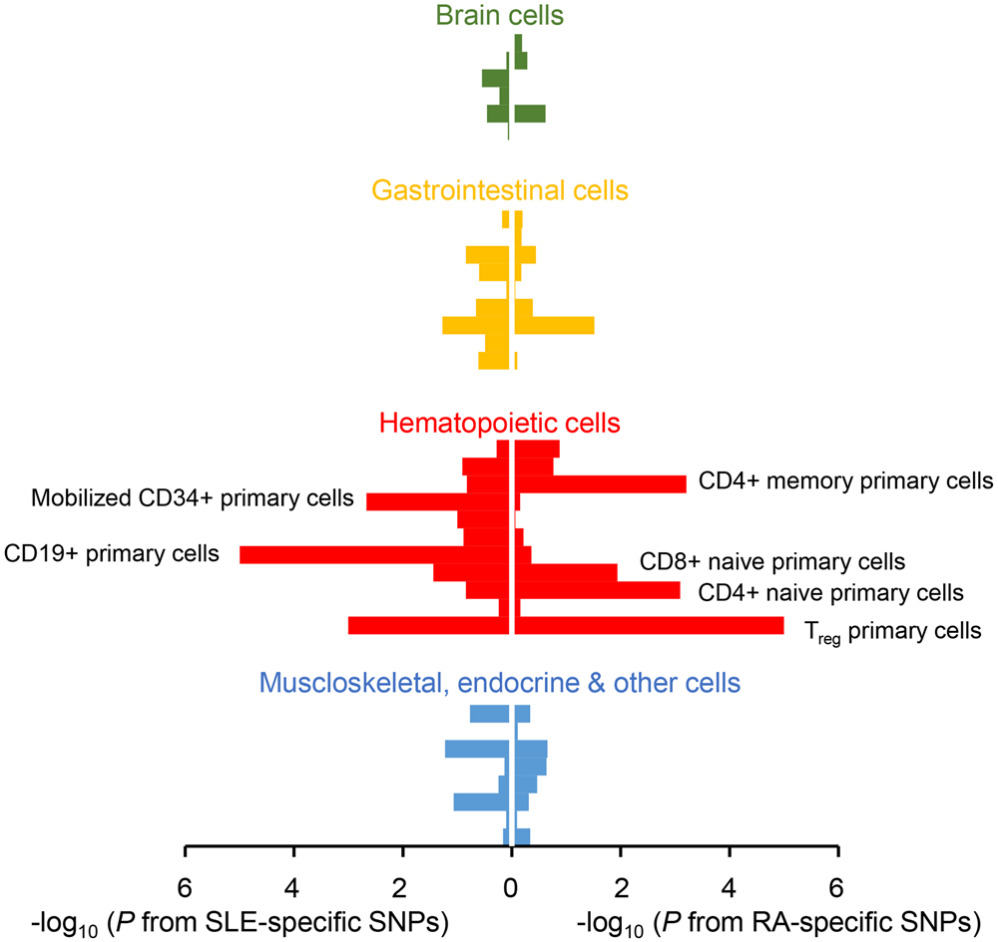
Significant overlaps between cell type-specific H3K4me3 histone modification marks and disease-specific variants. Enrichment *P* values of RA- and SLE-specific variants within H3K4me3 marks were plotted for each cell type in a minus log_10_ scale. The x-axis represents the transformed enrichment *P* values for SLE variants (left) and RA variants (right). The bars for each cell type were colored according to the lineage classification of the cell type. The names of tissues with enrichment *P* values < 0.05 were labeled next to the corresponding bar. The detailed information of this figure is shown in Supplementary Table S4.

The results showed that H3K4me3 peaks in regulatory T primary cells were significantly colocalized with RA- and SLE-specific SNPs (*P* < 1.0 × 10^−5^ and *P* = 9.9 × 10^−4^, respectively), although disease-specific SNPs were mutually exclusive between two diseases. This suggests that regulatory T cells may contribute to the RA and SLE pathogenesis via different genes and/or pathways, or via alternative expressional regulation of the same genes. In addition to regulatory T cells, other T-cell subtypes including CD4^+^ memory T primary cells and CD4^+^ naive T primary cells were implicated in RA by significant H3K4me3 localization on RA-specific SNPs (*P* ≤ 8.1 × 10^−4^). In SLE, CD19^+^ B primary cells and mobilized CD34^+^ primary cells showed significant enrichments (*P* < 1 × 10^−5^ and *P* = 2.14 × 10^−3^, respectively; Fig. 2, Supplementary Table S4).

**Figure 3:**
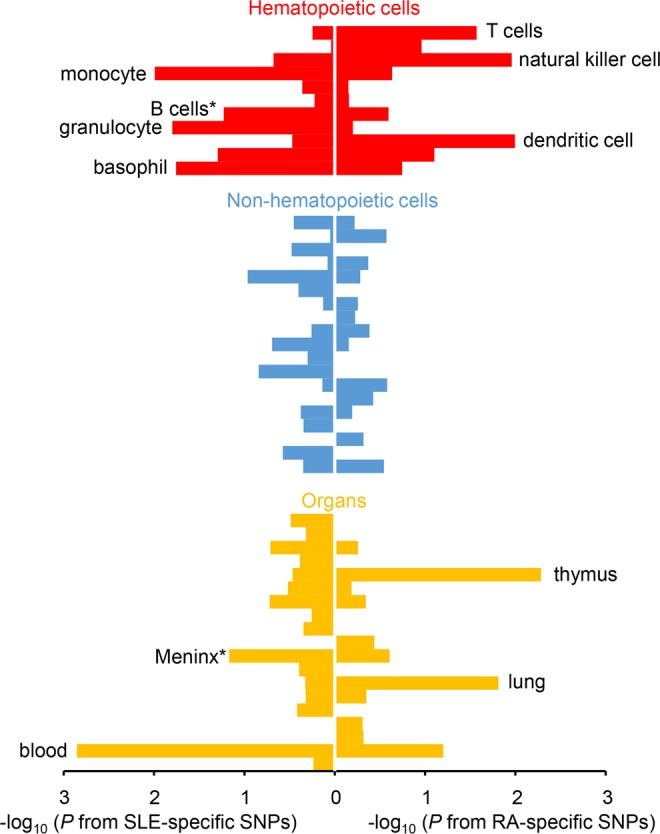
Significant overlaps between cell or organ type-specific enhancers and disease-specific variants. Enrichment *P* values of RA- and SLE-specific variants within enhancer regions were plotted for each cell or organ type in a minus log_10_ scale. The x-axis represents the transformed enrichment *P* values for SLE variants (left) and RA variants (right). Each cell or bar type were color categorized as hematopoietic cell, non-hematopoietic cell, or organ. The names of tissues or organs with enrichment *P* values < 0.05 were labeled next to the corresponding bar. Tissues or organs with *P* > 0.6 for both RA- and SLE-specific variants have been omitted in this figure. The detailed information of this figure is shown in Supplementary Table S5.

The disease-specific SNPs were evaluated for the enrichment in the cell-specific enhancer regions in various cell and organ types. We found significant overlap between RA-specific SNPs and enhancers in three immune cell types (*P* = 0.010 in dendritic cells; *P* = 0.011 in NK cells; *P* = 0.027 in T cells) and two organs (*P* = 0.0052 in thymus; *P* = 0.015 in lung; Fig. 3, Supplementary Table S5). These analyses consistently support the importance of T-cell biology in RA^33^ and the disease relevance of lung in RA. Lung is considered as a key organ in the initiation of RA-specific immune responses, especially for the production of anti-citrullinated protein antibodies (ACPA)^34^.

SLE-specific SNPs were significantly more in the enhancer regions in monocyte (*P* = 0.010), granulocyte (*P* = 0.016) and basophil (*P* = 0.018). We note the association of B cells was marginal (*P* = 0.059) in SLE. In an organ-enhancer analysis, the blood enhancer regions were significantly colocalized with the SLE-specific SNPs (*P* < 1.0 × 10^−4^; Fig. 3, Supplementary Table S5). The association of meninx related to psychiatric SLE approached the borderline of significance in this analysis (*P* = 0.068)^35^.

### Specific gene sets implicated by disease-specific alleles

It is plausible that genes in disease-specific loci are involved in disease-relevant biological pathways^36^. Therefore, we performed a gene-set enrichment analysis for genes near the top 1% of disease-specific SNPs using the DEPICT software^21^ and its own reconstituted gene sets. Many immune-related gene sets and some non-immune gene sets contained significantly more genes in disease-specific loci, with 236 pathways in RA and 199 pathways in SLE at false discovery rate (FDR) < 20% (Supplementary Tables S6 and S7).

T-cell-related gene sets were most highlighted in RA, including ‘abnormal CD8^+^ T-cell physiology (*P* = 9.68 × 10^−6^)’, ‘abnormal lymphocyte morphology (*P* = 3.06 × 10^−5^)’, and ‘increased T-cell number (*P* = 3.20 × 10^−5^)’. By contrast, other lymphocyte-related gene sets were most emphasized in SLE, including ‘glucose-6-phosphate isomerase protein-protein interaction (GPI PPI subnetwork, *P* = 2.08 × 10^−5^)’, ‘TRAF3 PPI subnetwork (*P* = 3.82 × 10^−5^)’ and ‘decreased interleukin-2 secretion (*P* = 4.57 × 10^−5^)’. The GPI PPI subnetwork is known as neuroleukin or lymphokine that induces antibody secretion of B-cells^37^. In addition, TRAF3 PPI subnetwork is involved in regulating B cell signaling and plasma cell development^38^. Many immune-related gene sets were shared in both RA and SLE, although their significance ranks varied (Supplementary Tables S6 and S7).

## Discussion

Our genome-wide approach showed that RA and SLE share highly similar genetic etiologies with some exceptional variants that may contribute to disease risk in a disease-specific manner. To evaluate the disease specificity of genetic associations between RA and SLE, we calculated the disease-specificity statistic *S* for each variant using disease association summary statistics in each disease and association heterogeneity statistics between two diseases. (We note that the statistic *S* is developed for a prioritization analysis but not for a parametric analysis. The statistic *S* dependent on MAFs of variants and sample sizes that largely affect standard errors of the disease effect size. Therefore, a null distribution of S is study-specific).

In our analyses, most of the disease-specific associations were explained by non-coding variants, suggesting that disease-specific variants have regulatory effects on regulatory annotations. As the regulatory regions such as histone modification and enhancers are highly cell and organ type-specific, we could trace which cell types or tissues are relevant to each disease by analyzing the overlap between regulatory regions and disease-specific variants. Additionally, genes within or near the disease-specifically associated loci could suggest which gene sets or biological pathways were associated with disease-specific outcomes.

In a series of the enrichment analyses using the top 1% of disease-specific variants in each disease, the importance of T cells in RA pathogenesis and B cells in SLE pathogenesis was identified by H3K4me3, This finding is supported by previous genetic and immunological studies^39–44^. Notably, regulatory T primary cells were identified in both diseases, although disease-specific variants did not overlap. This implicates that regulatory T cells may have a role in the pathogenesis of RA and SLE with their disease-specific pathways.

A permutation analysis for cell and organ type-specific enhancers, we detected additional cell types and tissues involved in RA and SLE pathogenesis. The significance values were relatively weak due to small coverage of the entire enhancer regions in the human genome that contained relatively few disease-specific SNPs. Enhancers with RA-specific variants supported a role for the antibody-mediated immune systems^45^, whereas enhancers with SLE-specific variants were significantly overlapped with enhancer regions in the monocytes that contribute to innate immunity and subsequent antigen presentation^46^. SLE-specific variants also were associated with granulocytes and basophils, which interact with B cells in SLE pathogenesis. Interestingly, we found the association of lung in RA, where ACPA may be produced in patients with RA^47,48^.

Gene set enrichment analyses using disease-specific variants indicated that the majority of pathways detected at FDR < 20% were related to immune response and cytokines. Most their effects were considered as valid in the cell types that were identified in the enrichment analysis for histone marks and enhancers (e.g., T cell-related gene sets in RA; B cell-related and immunoglobulin- related gene sets in SLE; lung inflammation in RA). These results were consistent with the enrichment results for cell- and organ specific enhancers.

In conclusions, this work illustrated the advantage of identifying disease-specific variants between two similar diseases in understanding disease-specific cell types, organ and biological pathways. We identified disease-specific variants between two rheumatic diseases, RA and SLE, and provided statistical evidence for disease-specific cells, tissues, and gene sets that may drive the distinctly different disease-specific phenotypes.

## Supplementary information

Supplementary Information

Supplementary Dataset
