# Supplementary material for: Genetic variants differentially associated with rheumatoid arthritis and systemic lupus erythematosus reveal the disease-specific biology
Source: Sci Rep. 2019 Feb 25;9:2739. doi: 10.1038/s41598-019-39132-2 (PMC6390106; doi:10.1038/s41598-019-39132-2)
Supplement: Supplementary file 1 — Supplementary Information [file 41598_2019_39132_MOESM1_ESM.pdf]

Supplementary Information

Genetic variants differentially associated with rheumatoid arthritis and systemic lupus erythematosus reveal the disease-specific biology

Jiwoo Lim, Kwangwoo Kim*

Department of Biology, Kyung Hee University, Seoul 02447, Republic of Korea

*Correspondence should be addressed to KK. (kkim@khu.ac.kr)

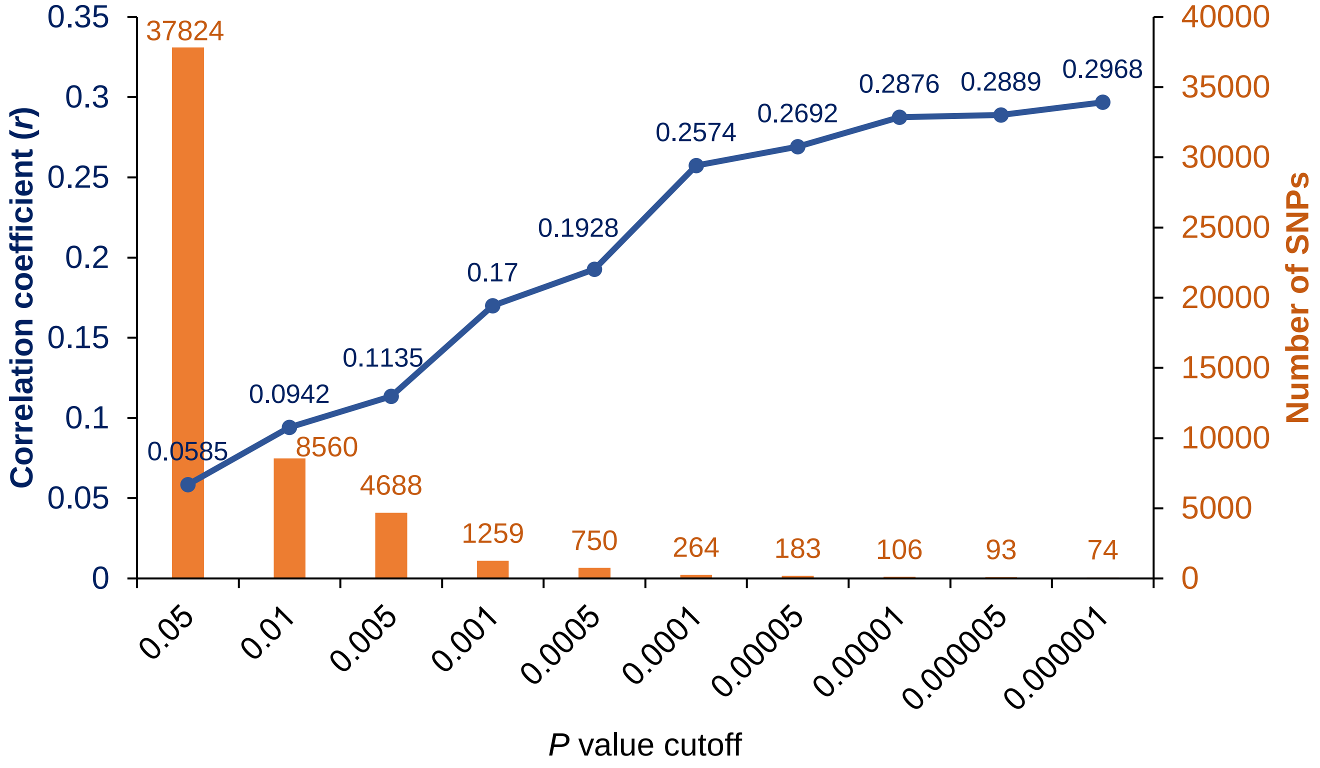

Supplementary Figure S1. Positive correlation coefficients of *P* values between rheumatoid arthritis (RA) and systemic lupus erythematosus (SLE). *P* values were transformed to the $\log_{10}P$ scale with a plus or minus sign representing the positive or negative effect of a minor allele). For the correlation tests, we extracted SNPs that pass a disease association *P* threshold in RA or SLE. The bars present the number of SNPs used in the correlation test and the blue line shows correlation coefficients *r* of transformed *P* values in RA and SLE. All observed correlation coefficients represent positive correlations, indicating that risk alleles are shared between RA and SLE. The correlation got stronger as the P threshold was more stringent.

Supplementary Table S1. Significant associations of 14 SNPs in a cross-disease meta-analysis but not a single-disease analysis.

					Meta-analysis				RA		SLE			Confirmed in a further analysis or other studies?	
SNP ID	CHR	Position	EA	NEA	OR	OR95L	OR95U	P	OR	P	OR	P	Location	RA	SLE
rs187339910	1	2523878	*A*	*G*	1.104	1.066	1.144	4.48E-08	1.10	1.80E-07	1.15	0.016	*MMEL1*	○ (ref [1])	-
rs10175798	2	30449594	*A*	*G*	1.093	1.065	1.122	1.72E-11	1.09	1.30E-07	1.11	2E-04	4.8kb 5' of *LBH*	○ (ref [2])	○ (ref [3])
rs668998	3	159715551	*A*	*G*	0.941	0.923	0.961	4.92E-09	0.94	0.00017	0.95	0.061	CTD-2049J23.2, *IL12A*	-	○ (ref [4])
rs77060203	5	55530306	*A*	*G*	1.096	1.063	1.131	8.71E-09	1.09	3.20E-07	1.11	0.001	1.1kb 5' of *ANKRD55*	○ (ref[2])	-
rs10814140*	9	34737018	*A*	*G*	0.930	0.911	0.949	5.75E-12	0.92	3.30E-07	1.01	0.808	*FAM205A*	○ (ref [2])	-
rs10821949	10	63811678	*T*	*C*	1.120	1.085	1.156	2.43E-12	1.11	5.80E-08	1.15	1.97E-06	*ARID5B*	○ (ref [2])	○ (ref [4])
rs3852411	10	64044999	*A*	*T*	1.158	1.102	1.216	6.68E-09	1.13	0.00014	1.2	6.58E-05	RP11-120C12.3, *RTKN2*	○ (ref [2])	○ (ref [4])
rs7927748	11	128499574	*T*	*G*	1.092	1.065	1.121	9.66E-12	1.08	5.90E-07	1.15	1.39E-06	RP11-744N12.3	○ (ref [2])	○ (ref [4])
rs4766578*	12	111904371	*A*	*T*	0.913	0.887	0.94	6.82E-10	0.93	1.30E-06	0.86	1.13E-07	*ATXN2* *SH2B3*	○ (ref [2])	○ (ref [4])
rs8045689	16	28988269	*T*	*C*	0.919	0.893	0.946	8.56E-09	0.92	3.10E-05	0.92	0.003	*SPNS1*	-	-
rs9308364	16	86003446	*T*	*C*	0.926	0.901	0.951	3.59E-08	0.95	0.00043	0.86	3.41E-07	47kb 3' of *IRF8*	○ (ref [2])	○ (ref [4])
rs901886	19	10402131	*T*	*C*	0.918	0.893	0.944	1.53E-09	0.94	2.30E-05	0.86	1.38E-07	*ICAM5*	○ (ref [2])	○ (ref [4])
rs74908652	19	10422972	*T*	*C*	1.135	1.09	1.181	5.37E-10	1.1	2.20E-05	1.23	3.08E-07	*FDX1L*	○ (ref [2])	○ (ref [4])
rs140496	22	21926456	*A*	*G*	0.92	0.894	0.948	2.62E-08	0.93	8.60E-05	0.88	3.52E-05	*UBE2L3*	○ (ref [2])	○ (ref [4])

* The SNPs are in LD with at least one nonsynonymous SNP ($r^2 > 0.8$; distance < 500kb).

1 Stahl, E. A. *et al.* Genome-wide association study meta-analysis identifies seven new rheumatoid arthritis risk loci. *Nat Genet* **42**, 508-514, doi:10.1038/ng.582 (2010).

2 Okada, Y. *et al.* Genetics of rheumatoid arthritis contributes to biology and drug discovery. *Nature* **506**, 376-381, doi:10.1038/nature12873 (2014).

3 Morris, D. L. *et al.* Genome-wide association meta-analysis in Chinese and European individuals identifies ten new loci associated with systemic lupus erythematosus. *Nat Genet* **48**, 940-946, doi:10.1038/ng.3603 (2016).

4 Bentham, J. *et al.* Genetic association analyses implicate aberrant regulation of innate and adaptive immunity genes in the pathogenesis of systemic lupus erythematosus. *Nat Genet* **47**, 1457-1464, doi:10.1038/ng.3434 (2015).

CHR=chromosome location, EA=effect allele, NEA=non-effect allele, OR=odds ratio, OR95L=95% confidence interval lower bound of odds ratio, OR95U=95% confidence interval upper bound of odds ratio, P=*P*-value, RA=rheumatoid arthritis, SLE=systemic lupus erythematosus.

Supplementary Table S4. Enrichments of disease-specific variants within H3K4me3 histone modification marks in various cell types.

Cell-type	*P* from RA-specific variants	*P* from SLE-specific variants	Subtype
Treg primary cells	$<1x10^{-5}$	$9.9x10^{-4}$	Hematopoietic
CD4+ memory primary cells	$6.2x10^{-4}$	0.149	Hematopoietic
CD4+ naive primary cells	$8.1x10^{-4}$	0.143	Hematopoietic
CD8+ naive primary cells	0.011	0.036	Hematopoietic
Mucosa, colon	0.030	0.053	Gastrointestinal
Mesenchymal stem cells (bone marrow)	0.133	0.524	Hematopoietic
CD8+ memory primary cells	0.173	0.124	Hematopoietic
Muscle satellite cultured cells	0.221	0.059	Muscloskeletal, endocrine & others
Mesenchymal stem cells (adipose)	0.232	0.730	Muscloskeletal, endocrine & others
Cingulate gyrus	0.241	0.350	Brain
Chondrocytes (mesenchymal stem cells)	0.344	0.569	Muscloskeletal, endocrine & others
Rectal smooth muscle	0.362	0.144	Gastrointestinal
Mucosa, duodenum	0.411	0.219	Gastrointestinal
CD19+ primary cells	0.437	$<1x10^{-5}$	Hematopoietic
Adipocyte (mesenchymal stem cells)	0.462	0.687	Muscloskeletal, endocrine & others
Skeletal muscle	0.463	0.171	Muscloskeletal, endocrine & others
Adult kidney	0.490	0.086	Muscloskeletal, endocrine & others
Mid-Frontal lobe	0.521	0.795	Brain
CD34+ primary cells	0.614	0.130	Hematopoietic
Stomach smooth muscle	0.637	0.661	Gastrointestinal
Substantia nigra	0.653	0.982	Brain
Smooth muscle, colon	0.667	0.918	Gastrointestinal
Mucosa, stomach	0.673	0.250	Gastrointestinal
CD3+ primary cells	0.702	0.572	Hematopoietic
Mobilized CD34+ primary cells	0.703	0.002	Hematopoietic
Pancreatic islets	0.781	0.992	Muscloskeletal, endocrine & others
Adult liver	0.795	0.243	Gastrointestinal
Adipose nuclei	0.803	0.784	Muscloskeletal, endocrine & others
CD34+ cultured cells	0.865	0.100	Hematopoietic
Mucosa, rectum	0.866	0.792	Gastrointestinal
Inferior temporal lobe	0.904	0.283	Brain
Anterior caudate	0.984	0.846	Brain
Duodenum smooth muscle	0.991	0.324	Gastrointestinal
Hippocampus middle	0.993	0.590	Brain

Supplementary Table S5. Enrichments of disease-specific variants within enhancer in various cell and organ types.

Cell or organ type	*P* from RA-specific variants	*P* from SLE-specific variants	Classification
thymus	0.005	0.342	Organ
dendritic cell	0.010	0.339	Hematopoietic cell-types
natural killer cell	0.011	0.210	Hematopoietic cell-types
lung	0.015	0.471	Organ
T cell	0.027	0.567	Hematopoietic cell-types
blood	0.063	0.001	Organ
circulating cell	0.079	0.051	Hematopoietic cell-types
neutrophil	0.109	0.913	Hematopoietic cell-types
basophil	0.179	0.018	Hematopoietic cell-types
monocyte	0.231	0.010	Hematopoietic cell-types
meninx	0.248	0.068	Organ
lymphocyte of B lineage	0.253	0.059	Hematopoietic cell-types
fibroblast of gingiva	0.262	0.730	Non hematopoietic cell-types
stromal cell	0.267	0.891	Non hematopoietic cell-types
acinar cell	0.285	0.447	Non hematopoietic cell-types
prostate gland	0.364	1.000	Organ
epithelial cell of esophagus	0.375	1.000	Non hematopoietic cell-types
hepatocyte	0.414	0.553	Non hematopoietic cell-types
skin fibroblast	0.426	0.833	Non hematopoietic cell-types
liver	0.446	0.478	Organ
spleen	0.452	0.190	Organ
blood vessel	0.480	1.000	Organ
cardiac myocyte	0.481	1.000	Non hematopoietic cell-types
eye	0.491	1.000	Organ
respiratory epithelial cell	0.521	0.109	Non hematopoietic cell-types
tonsil	0.553	0.194	Organ
keratinocyte	0.555	0.742	Non hematopoietic cell-types
intestinal epithelial cell	0.594	1.000	Non hematopoietic cell-types
fibroblast of choroid plexus	0.601	1.000	Non hematopoietic cell-types
tendon cell	0.602	0.352	Non hematopoietic cell-types
granulocyte	0.628	0.016	Hematopoietic cell-types
ciliated epithelial cell	0.640	0.421	Non hematopoietic cell-types
pericyte cell	0.647	1.000	Non hematopoietic cell-types
testis	0.650	0.304	Organ
fibroblast of tunica adventitia of artery	0.655	0.799	Non hematopoietic cell-types
mesothelial cell	0.664	0.800	Non hematopoietic cell-types
enteric smooth muscle cell	0.666	1.000	Non hematopoietic cell-types
lens epithelial cell	0.674	1.000	Non hematopoietic cell-types
heart	0.679	1.000	Organ
macrophage	0.695	0.598	Hematopoietic cell-types
hair follicle cell	0.697	0.203	Non hematopoietic cell-types
smooth muscle cell of prostate	0.698	1.000	Non hematopoietic cell-types
mast cell	0.703	0.437	Hematopoietic cell-types

astrocyte	0.709	0.864	Non hematopoietic cell-types
urothelial cell	0.728	0.860	Non hematopoietic cell-types
epithelial cell of Malassez	0.736	0.858	Non hematopoietic cell-types
myoblast	0.788	0.623	Non hematopoietic cell-types
vascular associated smooth muscle cell	0.788	0.906	Non hematopoietic cell-types
epithelial cell of prostate	0.806	1.000	Non hematopoietic cell-types
mammary epithelial cell	0.810	0.912	Non hematopoietic cell-types
large intestine	0.853	1.000	Organ
preadipocyte	0.881	0.845	Non hematopoietic cell-types
gingival epithelial cell	0.895	0.807	Non hematopoietic cell-types
fat cell	0.901	1.000	Non hematopoietic cell-types
sensory epithelial cell	0.915	0.970	Non hematopoietic cell-types
blood vessel endothelial cell	0.940	0.917	Non hematopoietic cell-types
skeletal muscle cell	0.944	0.679	Non hematopoietic cell-types
brain	0.953	0.959	Organ
mesenchymal cell	0.965	0.961	Non hematopoietic cell-types
kidney epithelial cell	0.996	0.397	Non hematopoietic cell-types
amniotic epithelial cell	1.000	0.787	Non hematopoietic cell-types
bronchial smooth muscle cell	1.000	1.000	Non hematopoietic cell-types
cardiac fibroblast	1.000	0.267	Non hematopoietic cell-types
chondrocyte	1.000	0.451	Non hematopoietic cell-types
corneal epithelial cell	1.000	1.000	Non hematopoietic cell-types
endothelial cell of hepatic sinusoid	1.000	0.739	Non hematopoietic cell-types
endothelial cell of lymphatic vessel	1.000	1.000	Non hematopoietic cell-types
fibroblast of lymphatic vessel	1.000	0.144	Non hematopoietic cell-types
fibroblast of periodontium	1.000	0.704	Non hematopoietic cell-types
fibroblast of pulmonary artery	1.000	0.500	Non hematopoietic cell-types
fibroblast of the conjuctiva	1.000	1.000	Non hematopoietic cell-types
hepatic stellate cell	1.000	1.000	Non hematopoietic cell-types
iris pigment epithelial cell	1.000	1.000	Non hematopoietic cell-types
keratocyte	1.000	1.000	Non hematopoietic cell-types
melanocyte	1.000	0.833	Non hematopoietic cell-types
neuron	1.000	1.000	Non hematopoietic cell-types
neuronal stem cell	1.000	1.000	Non hematopoietic cell-types
osteoblast	1.000	0.911	Non hematopoietic cell-types
placental epithelial cell	1.000	1.000	Non hematopoietic cell-types
reticulocyte	1.000	1.000	Non hematopoietic cell-types
retinal pigment epithelial cell	1.000	1.000	Non hematopoietic cell-types
smooth muscle cell of the esophagus	1.000	1.000	Non hematopoietic cell-types
smooth muscle cell of trachea	1.000	0.333	Non hematopoietic cell-types
trabecular meshwork cell	1.000	1.000	Non hematopoietic cell-types
uterine smooth muscle cell	1.000	0.626	Non hematopoietic cell-types
adipose tissue	1.000	0.581	Organ
esophagus	1.000	1.000	Organ
female gonad	1.000	1.000	Organ
gallbladder	1.000	0.384	Organ

internal male genitalia	1.000	1.000	Organ
kidney	1.000	1.000	Organ
lymph node	1.000	0.404	Organ
olfactory region	1.000	1.000	Organ
pancreas	1.000	1.000	Organ
parotid gland	1.000	1.000	Organ
penis	1.000	1.000	Organ
placenta	1.000	1.000	Organ
salivary gland	1.000	1.000	Organ
skeletal muscle tissue	1.000	0.452	Organ
skin of body	1.000	1.000	Organ
small intestine	1.000	1.000	Organ
smooth muscle tissue	1.000	1.000	Organ
spinal cord	1.000	0.555	Organ
stomach	1.000	1.000	Organ
submandibular gland	1.000	1.000	Organ
throat	1.000	1.000	Organ
thyroid gland	1.000	1.000	Organ
tongue	1.000	0.413	Organ
umbilical cord	1.000	1.000	Organ
urinary bladder	1.000	1.000	Organ
uterus	1.000	0.481	Organ
vagina	1.000	0.326	Organ
